# Extensive phenotypic plasticity of a Red Sea coral over a strong latitudinal temperature gradient suggests limited acclimatization potential to warming

**DOI:** 10.1038/srep08940

**Published:** 2015-03-10

**Authors:** Yvonne Sawall, Abdulmoshin Al-Sofyani, Sönke Hohn, Eulalia Banguera-Hinestroza, Christian R. Voolstra, Martin Wahl

**Affiliations:** 1 Helmholtz Center for Ocean Research (GEOMAR), Wischhofstr. 1-3, 24148 Kiel, Germany; 2 Faculty of Marine Science, King Abdulaziz University (KAU), P.O. Box 80207, Jeddah 21589, Saudi Arabia; 3 Ecological Modelling, Leibniz Center for Tropical Marine Ecology (ZMT), Fahrenheitstr. 6, 28359 Bremen, Germany; 4 Red Sea Research Center, King Abdullah University of Science and Technology (KAUST), Thuwal 23955-6900, Saudi Arabia

## Abstract

Global warming was reported to cause growth reductions in tropical shallow water corals in both, cooler and warmer, regions of the coral species range. This suggests regional adaptation with less heat-tolerant populations in cooler and more thermo-tolerant populations in warmer regions. Here, we investigated seasonal changes in the *in situ* metabolic performance of the widely distributed hermatypic coral *Pocillopora verrucosa* along 12° latitudes featuring a steep temperature gradient between the northern (28.5°N, 21–27°C) and southern (16.5°N, 28–33°C) reaches of the Red Sea. Surprisingly, we found little indication for regional adaptation, but strong indications for high phenotypic plasticity: Calcification rates in two seasons (winter, summer) were found to be highest at 28–29°C throughout all populations independent of their geographic location. Mucus release increased with temperature and nutrient supply, both being highest in the south. Genetic characterization of the coral host revealed low inter-regional variation and differences in the *Symbiodinium* clade composition only at the most northern and most southern region. This suggests variable acclimatization potential to ocean warming of coral populations across the Red Sea: high acclimatization potential in northern populations, but limited ability to cope with ocean warming in southern populations already existing at the upper thermal margin for corals.

As a consequence of climate change due to increased emissions of greenhouse gases, sea surface temperatures (SST) have risen by ~0.7°C since the 1950s and are expected to increase further by 0.2°C per decade in tropical seas^1^. Growth of shallow water corals (*Porites spp.*) in the Indo-Pacific was found to positively correlate with temperature along latitudinal gradients^2^, most likely due to higher metabolic activity at warmer temperatures. In both, colder and warmer, regions of a species' range, however, global warming was repeatedly found to reduce coral growth independently of temperature history^3^,^4^,^5^, a finding suggesting adaptation of the coral holobiont (coral and/or symbionts) to local thermal regimes^6^. A reduction of coral growth can have far-reaching ecological consequences given that scleractinian corals are the main bioengineers in modern coral reefs providing a structurally complex habitat for numerous species^7^.

Reasons for reduced coral growth at temperatures above normally experienced temperatures is likely found in a shortage of energy for calcification. This may happen when the coral's energy supplying symbionts^8^,^9^, dinoflagellates of the genus *Symbiodinium* (zooxanthellae), are damaged and reduced in numbers under temperature stress^10^ and/or when energy is compromised for stress-preventing processes, such as the expression of heat-shock proteins^11^. On the other hand, however, it was found that some coral species seem to be able to adapt to environmental changes (including warming) by changing their *Symbiodinium* composition to more robust types^12^,^13^,^14^ or by a rather fast genetic adaptation of a given *Symbiodinium* type^15^, which is rather promising outlook in the context of global warming. Furthermore, corals thriving in regions with naturally high water temperatures (>31°C) may serve as a ‘genetic reservoir' of temperature resistant corals possibly able to populate other geographic regions with lower but increasing water temperatures. For this reason, it is of particular importance to investigate underlying mechanisms of thermo-tolerant corals, which will help us to better understand and predict the future of coral reefs.

An ideal system to study the coral's mechanisms to adjust to different prevailing temperature regimes is the Red Sea. The Red Sea is characterized by a strong temperature gradient with particularly high temperatures in the south (>31.5°C during several months in summer^16^; Fig. 1a). Parallel to the temperature gradient, the depletion of productive Gulf of Aden waters entering the Red Sea drives a strong nutrient gradient, as indicated by high chlorophyll *a* (chl *a*) concentrations in the south and low chl *a* concentrations in the northern and central Red Sea (Fig. 1b). This latitudinal pattern prevails all year except in winter, when deep mixing replenishes surface layer nutrients in the northern Red Sea and Gulf of Aqaba (Fig. 1b). While high nutrient loads can be detrimental to coral reef ecosystems by promoting growth of macroalgae that compete with corals for light and space^7^, increased nutrient concentrations can be beneficial at the organismal level by enhancing the coral's nutritional and energetic status resulting in increased coral growth (tissue and skeleton)^17^,^18^. The combined effect of increasing temperature and nutrients on corals, however, seems to be species specific^19^ and depended on the quality of nutrients^20^. Furthermore, corals featuring a high flexibility in nutrients and energy acquisition (autotrophy versus heterotrophy) overcome thermal stress more likely^21^,^22^. Despite a wide range of environmental conditions (including extremes) throughout the Red Sea, coral diversity and reef complexity remain high^23^. The occurrence of certain coral species throughout this highly differentiated gradient suggests regional adaptation. The Red Sea, thus, constitutes a unique natural laboratory to study the effect of different temperature and nutrient regimes *in situ*, including conditions considered detrimental in other geographic regions under global change scenarios^24^.

Here, we investigated the physiological performance and genetic composition of the widely distributed coral species *Pocillopora verrucosa* (Ellis and Solander, 1786) in a rare large scale *in situ* study spanning 12 degrees of latitude (i.e. 2,000 km coast line) and including steep environmental gradients (i.e. over 6°C annual mean temperature), as well as two seasons (i.e. summer of September 2011 and winter of March 2012). Special emphasis was given to calcification, as the fundamental process of reef growth and hence the formations of complex and diverse coral reef systems. Additionally, photosynthetic rates (as the primary energy source) and mucus release rates (as a major energy loss), as well as the tissue composition (for nutritional status and energy reserves) were assessed, related to environmental conditions and further to calcification. For our study, we hypothesized that coral populations are adapted to prevailing local conditions, which should be reflected in a low latitudinal pattern in coral performance and a differentiation in the genetic composition from north to south.

## Methods

### Study sites

**Figure 1:**
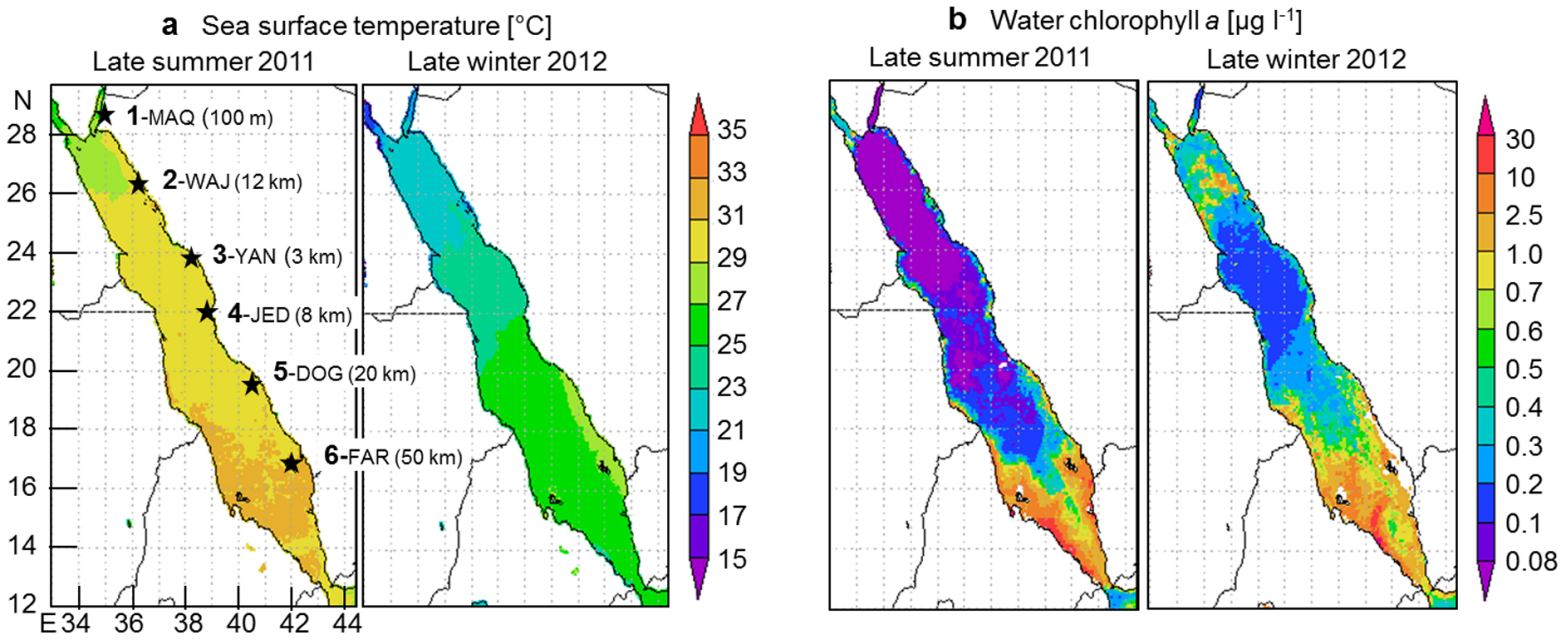
Map with study sites and environmental conditions. (a) sea surface temperature and (b) water chlorophyll *a* concentrations as a proxy for nutrient supply were derived from NASA, Giovanni online data system, developed and maintained by the NASA GES DISC, Ocean Color Radiometry, monthly averaged MODIS-Aqua 4 km. Images represent averaged data from July to September (temperature) or to October 2011(chlorophyll *a*, late summer) and from January to March 2012 (late winter). Study sites are indicated in a) with 1-MAQ = Maqna, 2-WAJ = Al-Wajh, 3-YAN = Yanbu, 4-JED = Jeddah, 5-DOG = Doga and 6-FAR = Farasan Islands, and the distance from the coast is indicated in brackets.

Six reefs were chosen along the Saudi Arabian Red Sea coast spanning over 12 degrees of latitude and being separated by ~300 km between each reef (Fig. 1a). They covered a large range of environmental conditions, as previously described by Sawall, et al.^16^: Temperature varied between 21°C–27°C (winter – summer) in the north (1-MAQ) and 28°C–33°C in the south (5-DOG, 6-FAR), and water chlorophyll *a* (chl *a*) concentrations varied between 0.04–0.26 μg chl *a* l^−1^ (summer – winter) in the north (1-MAQ) and 1.45–2.73 μg chl *a* l^−1^ (winter – summer) in the south (6-FAR). Light intensities in 5 m depth ranged between 33 and 44 E m^−2^ d^−1^ photosynthetic active radiation (PAR) throughout most part of the Red Sea in September 2011 and March 2012, but dropped down to ~20 E m^−2^ d^−1^ PAR at the most southern site 6-FAR in summer^16^. All reefs were at least 3 km away from the coast in order to minimize land-based influences (Fig. 1a). One exception is given in the most northern region, the Gulf of Aqaba, where only rather narrow fringing reefs exist along the coast (Fig. 1a). Here, however, the study site 1-MAQ was more than 10 km from the next small village Maqna. All investigations were carried out at the wave-exposed western sites of the reefs at the reef edge or upper reef slope in 5 m depth.

### Metabolic rates

*In situ* incubations were conducted to measure calcification, photosynthesis, respiration and mucus release rates. The experimental design and *in situ* incubation setup were described previously for a parallel study on *P. verrucosa* zooxanthellae physiology^16^. Briefly, prior to incubations for metabolism measurements, 6 coral fragments from 5 m depth were chiseled off from the central part of 6 colonies (1 fragment per colony) at each study site. Each fragment was glued to a plastic screw and fixed in the reef for acclimatization. At two consecutive days, three fragments per day were individually placed in incubation chambers (plus one coral-free control chamber) at the experimental depth from 0800/0900 to 1600/1700 hrs. The incubation setup (constructed at GEOMAR) consisted of 4 cylindrical acrylic chambers (volume: 950 ml) containing a battery-run stirrer, a water inlet and outlet with one-way valves in front and behind the chamber respectively, a fragment holder and an oxygen sensor. Oxygen sensors were connected to a battery-run data logger, as well as an underwater PAR sensor installed next to the chambers. Each water inlet was connected to a programmable battery-run pump via tubing, which pumped the surrounding water through the chambers every 45 min for 2 min to flush the chambers. Each incubation intervals was 43 min long leading to about 9 incubation intervals per day, 8 incubation intervals were conducted during light conditions, 1 incubation interval during dark conditions in the morning (chambers were covered for that).

During incubations oxygen concentration and PAR were logged every minute. Photosynthesis rates derived from oxygen production rates were related to the corresponding PAR intensities (P-I curves). 3–4 times a day samples for total alkalinity (TA) measurement were extracted to calculate calcification rates. For this, 100 ml of water was sampled manually with a syringe at the chamber water outlet in the beginning and at the end of an incubation interval. Immediately after surfacing, water samples were filtered through a GF/F filter into 50 ml falcon tubes and poisoned with HgCl_2_. Later, TA was measured in duplicates via potentiometric titration with an automated titrator (Titroline 7000, SI Analytics, Germany) using 25 ml of sample and 0.05 M HCl (precision ±1%). TA was calculated using the Gran approximation by determining the second endpoint of the titration curve^25^, and the difference in TA of the initial and final sample was used to calculate the calcification rate^26^. Calcification rates were related to the corresponding PAR intensities (C-I curves).

Respiration rates and dark calcification rates were derived from dark incubations. Respiration rates were added to the P-I curves. Dark calcification rates were added to the C-I curves, however corresponding to 100 μE m^−2^ s^−1^ (instead of 0) PAR in order to account for the delayed adjustment of calcification rates to dark conditions of about 25 min^27^. PAR intensity prior the dark incubation was 200–300 μE m^−2^ s^−1^.

A separate incubation setup was used to measure the rate of mucus release. These chambers (n = 4) consisted of acrylic cylinders (950 ml volume) equipped with a stirrer, a coral fragment holder and a Teflon membrane ‘window' for gas exchange. They were deployed in the experimental site and filled with surrounding water. Three chambers were equipped with a coral fragment each and 1 chamber was left as a coral-free control. Incubations ran from 0900 to 1600 hrs. Three initial water samples of 1 L were collected prior to the incubations in the vicinity of the chambers and final water samples were collected at the end of the incubation period from each incubation chamber. The water samples were kept in cool boxes and filtered through pre-weight GF/F filters in the evening of the same day. The filters were dried (60°C) until constant weight and the carbon content was measured with a CN analyzer (Flash 2000, Thermo Scientific, USA, calibrated with Acetanilid) from the initial (n = 3) and final water samples (n = 1 of each chamber). Mucus release was expressed as the differences of carbon between the initial and final samples (minus the control). Only particulate carbon was measured assuming negligible rates of dissolved mucus release in *Pocillopora* species of the Red Sea^28^.

For the metabolic rates, gross oxygen production (photosynthesis) and consumption (respiration) rates were converted into carbon units assuming the metabolic quotients 1.1 for photosynthesis and 0.8 for respiration^9^,^29^. Calcification and mucus release were measured as carbon precipitation (CaCO_3_) and carbon release (particulate carbon), respectively. Daily rates of photosynthesis and calcification were integrated from reconstructed diurnal photosynthesis-irradiance (P-I) and calcification-irradiance (C-I) curves (Supplementary Information Fig. S1). Daily rates of mucus release were calculated assuming a 75% reduction of mucus release rates during the night and a 12:12 h day:night cycle^28^. Daily rates of respiration were calculated assuming enhanced respiration of 58% during the day^30^. For detailed description and equations for the calculation of daily metabolic rates see Supplementary Information.

### Coral tissue parameters

In order to assess the effects of nutrient availability and temperature on the nutritional and energetic status of the coral, which may further influence coral growth, the coral tissue was analyzed. For this, the tissue was removed from the skeleton with filtered seawater and an air gun. The resulting tissue slurry was homogenized (T18 basic Ultra Turrax, 10 s, 15,000 U min^−1^), aliquoted and frozen at −20°C for biomass and protein determination and at −80°C for lipid analyses. Biomass was measured as the tissue dry weight, the protein concentration was determined photometrically (DU 650 Spectrophotometer, Beckman, USA) using the BCA Protein Assay kit (Thermo Scientific, Rockford), and the lipid concentration was determined gravimetrically after extraction with chloroform:methanol^31^. Data of zooxanthellae pigmentation (photo-collecting pigments cm^−2^) were derived from a parallel study of Sawall, et al.^16^ on zooxanthellae of the same coral specimen. Also coral surface areas were derived from the parallel study (determined by the wax-coating method^16^;) and used to standardize metabolic rates, mucus release and tissue parameters.

### Genetic characterization

DNA extraction of the coral tissue from the same coral fragments used for the metabolism measurements was performed using a DNeasy Plant Mini Kit (Qiagen). A fragment of 564 bp was amplified for the *mtORF* region as described by Flot, et al.^32^. Sequencing was done in forward and reverse directions and aligned using MUSCLE^33^ available within the program MEGA V.5^34^. Alignment was performed with the default settings. Data of the abundance of dominant *Symbiodinium* clades were derived from Sawall, et al.^16^, summarized by the main clades and graphically opposed to the corresponding haplotype data for a direct comparison.

### Data analyses

Analyses of covariance (ANCOVA) was conducted to differentiate between the effects of season (categorical predictor) latitude (continuous predictor) and season*latitude (interacting effect) on the different metabolic rates and tissue composition, after testing for normality (Levene's test) and data transformation (Box-Cox) where necessary. For this, the ‘homogeneity-of-slope model' was used in STATISTICA 8 (Statsoft). Multiple regression analyses were conducted to test relationships between each of the response variables biomass, protein content, lipid content, pigmentation and mucus release and the possible explanatory variables temperature and nutrient availability (STATISTICA 8). Data on nutrient availability were derived from Sawall, et al.^16^, expressed as ‘water chl a*relative water flow' as an indicator for nutrient influx. To assess the effect of temperature and different physiological features on the calcification rates, multivariate statistic namely the distant based linear model (DistLM) was applied, since the predictor variables are not always independent of each other and partly correlate with each other. For this, a resemblance matrix based on Bray-Curtis similarity was compiled and the step-wise forward procedure with 999 permutations and the force inclusion of temperature was applied using the software Primer +PERMANOVA [PERMANOVA+ for PRIMER: Guide to software and statistica methods. Anderson, Gorley & Clarke 2008].

Coral host haplotype data from the 6 regions (N = 79) were analysed by a hierarchical Analyses of Molecular Variance (AMOVA) in order to determine the percentage of variance explained by inter- or intra-regional variation. The principal is that the total variance is partitioned into covariance components according to inter- and intra-regional population differences, which are then used to compute the fixation index (*F*-statistics). In our case the *F*-statistics calculates the variation between the regional and total variation (*F_st_*), for which we used Arlequin v. 3.5.1.3^35^, taking into account the number of mutations between haplotypes. The number of haplotypes in our samples was identified using the program DnaSP v. 5.^36^. The phylogenetic position of our samples as members of *P. verrucosa* was corroborated building a Maximum likelihood tree in MEGA V.5 34, which included all haplotypes identified in our samples together with those haplotypes from *Pocillopora* deposited in the Genbank data base (Pinzon et al. 2013; http://www.ncbi.nlm.nih.gov/).

## Results

### Metabolic rates

**Figure 2:**
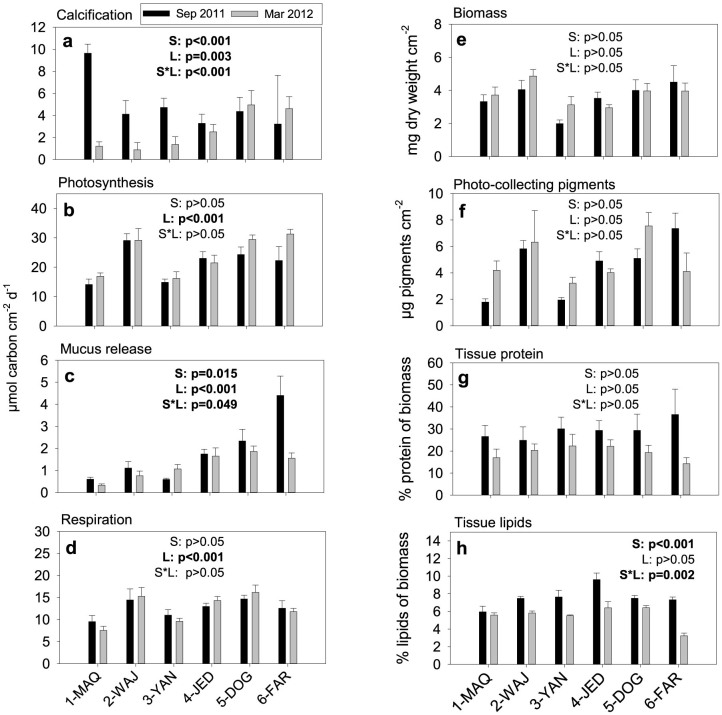
Metabolic rates (a–d) and tissue composition (e–h) of *P. verrucosa* from north (1-MAQ) to south (6-FAR) during summer (black, Sep 2011) and winter (grey, Mar 2012). Mean ± SE. Statistical results of Analysis of Covariance (ANCOVA) are presented within graphs, with S = effect of Season, L = effect of Latitude and S*L = interacting effect of Season and Latitude. Significant results are in bold.

**Figure 3:**
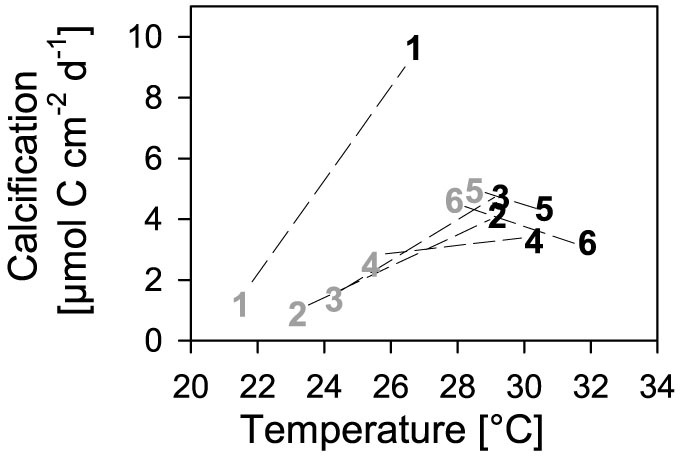
Calcification rates versus temperature. Numbers represent the mean calcification rates at prevailing temperatures for each site from north (1) to south (6) in summer (black, Sep 2011) and winter (grey, Mar 2012). The corresponding standard errors are presented in Fig. 2a. The dashed lines connect the values of the two seasons within each site.

**Figure 4:**
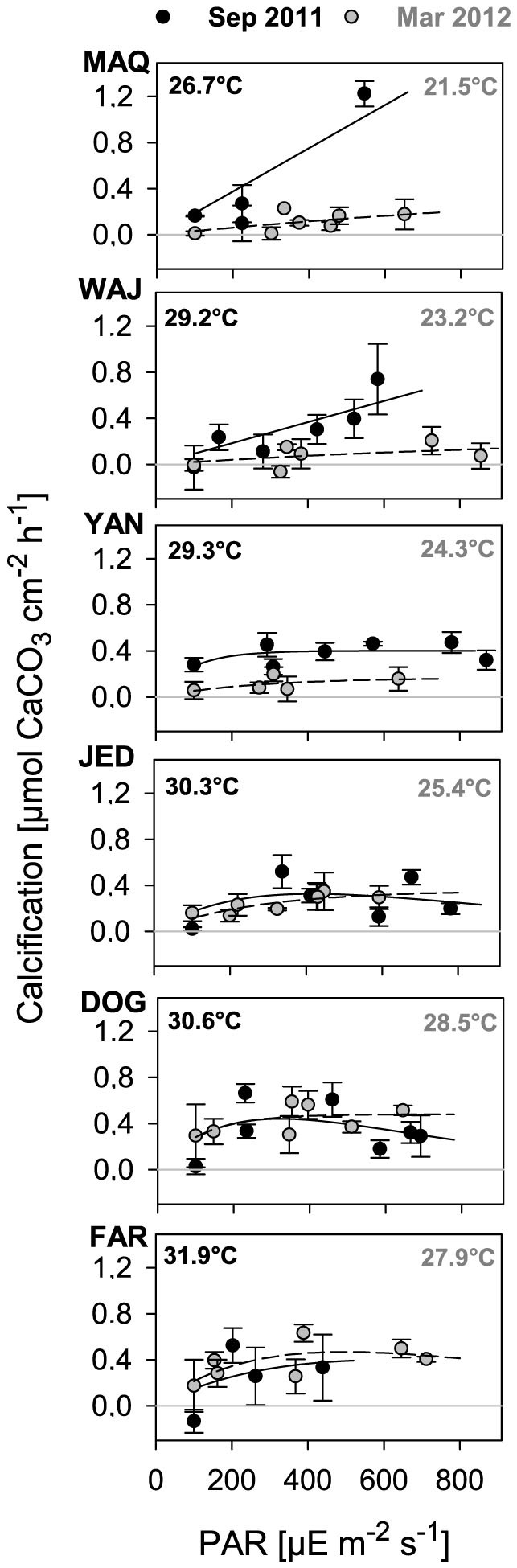
Calcification-Irradiance (C-I) curves at all sites in summer (black, Sep 2011) and winter (grey, Mar 2012). Mean ± SE (n = 3–6). Mean monthly temperatures were obtained by temperature loggers deployed over one year at the experimental site and depth^23^.

Daily calcification rates generally ranged between 0.9 ± 0.6 and 5.0 ± 1.3 μmol carbon cm^−2^ d^−1^ with one exception found in summer at the most northern site 1-MAQ, where the calcification rate was 9.7 ± 0.8 μmol carbon cm^−2^ d^−1^ (Fig. 2a). The calcification rates showed a strong and seasonally inverse latitudinal pattern (Fig. 2a). In summer, calcification rates were higher in the northern Red Sea, while they were higher in the southern Red Sea in winter (Fig. 2a). When calcification rates are plotted against temperatures, a strong temperature dependency of calcification across all sites is indicated (Fig. 3). Furthermore, the observed dependency indicates a temperature optimum between 28 and 29°C with calcification rates of ~4.5 μmol carbon cm^−2^ d^−1^ in the main Red Sea and a 2-fold higher rate at 1-MAQ, Gulf of Aqaba, (Fig. 3); independent whether this is the maximum (northern regions) or minimum (southern regions) temperature locally experienced. C-I curves generally revealed an increase of calcification with light intensity, albeit with a few exceptions (Fig. 4; see also C-I compared to P-I curves, Supplementary Information Fig. S2). These exceptions were visible at 4-JED and 5-DOG in summer, where calcification decreased at midday, coinciding with concomitantly high temperatures (>30°C) and high light intensities (>500 μE m^−2^ s^−1^) (Fig. 4).

Daily gross photosynthesis rates (net photosynthesis + respiration (respiration in Fig. 2d)) increased from north to south particularly in winter (except 2-WAJ), leading to 2-fold higher rates at 6-FAR (31.3 ± 1.8 μmol carbon cm^−2^ d^−1^), compared to 1-MAQ (16.9 ± 1.2; Fig. 2b). In contrast to that, there was no significant seasonal variation throughout sites, however a tendency towards lower photosynthetic rates during summer in the south (30% reduction at 6-FAR; Fig. 2b).

Daily mucus release rates revealed a particularly strong latitudinal trend in summer, where it increased more than 5-fold from north (1-MAQ: 0.6 ± 0.1 μmol carbon cm^−2^ d^−1^) to south (6-FAR: 4.4 ± 0.9) (Fig. 2c). In winter, this trend was less pronounced and mucus release rates were generally lower compared to summer (Fig. 2c). Furthermore, mucus release was significantly and positively related to increasing temperature as well as to nutrient availability (Tab. 1).

### Tissue composition

Biomass varied between 2.0 ± 0.2 (3-YAN summer) and 4.9 ± 0.4 mg tissue dry weight cm^−2^ (2-WAJ winter) and zooxanthellae pigmentation varied between 1.8 ± 0.1 (1-MAQ summer) and 9.6 ± 0.4 μg photo-collecting pigments cm^−2^ (5-DOG winter). Both parameters, however, did not follow a latitudinal or seasonal pattern (Fig. 2e,f) and could consequently also not be related to changes in nutrient availability or temperature (multiple regression, Tab. 1). The protein content of the tissue was slightly higher in summer compared to winter (not significant) and rather constant throughout the sites. Minimum and maximum values were found at the most southern sites 6-FAR, with 36.5 ± 11.5% in summer and 14.2 ± 2.8% in winter (Fig. 2g). The lipid content was also generally higher in summer than in winter and varied between 3.2 ± 0.3% (6-FAR winter) and 9.6 ± 0.7 (4-JED summer) (Fig. 2h). Both, protein and lipid content, correlated positively with temperature and negatively with nutrient availability (Tab. 1).

### Relationship between calcification and biological parameters

The potential effect of biogenic controls on calcification additionally to the temperature effect was tested by multiple regression (DistLM; results visualized by distance based redundancy analysis [dbRDA] as Supplementary Information Fig. S3). When testing across season and latitude, it was found that only temperature explained a significant and substantial amount of the variation in calcification (50%, Tab. 2, Fig. S3). When separating by season, it was found that in summer the protein content of the biomass and the photosynthetic rates explained an additional ~26% to temperature (72%) of the variation in calcification between sites (Tab. 2). In contrast, in winter, again only temperature explained a significant part (83%) of the variation in calcification between sites.

### Genetic composition

**Figure 5:**
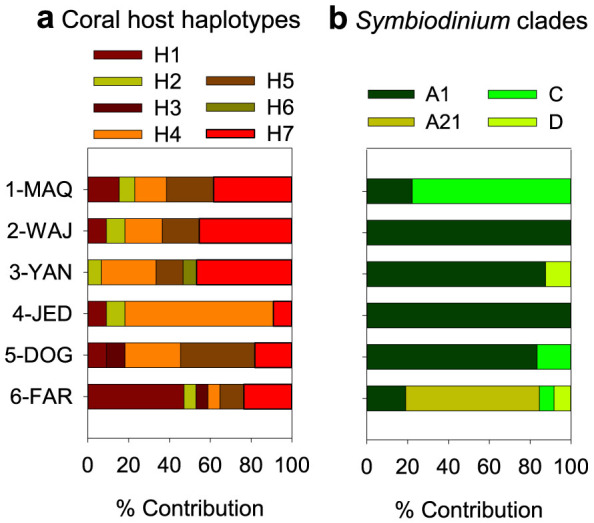
Genetic composition of coral host (a) and zooxanthellae (b). (a) 7 haplotypes of *P. verrucosa* (H1–H7) were found based on the mitochondrial marker known as an ‘ORF of unknown function'. (b) Summarized *Symbiodinium* compositions of dominant ITS2-type clades. Detailed *Symbiodinium* composition is presented in Sawall, et al.^16^.

A total of 7 haplotypes were identified based on the *mtORF* region, most of them present throughout the Red Sea (Fig. 5a). The genetic divergence between the regions is rather small with *F*_ST_ = 0.091 (p = 0.015), if followed the classification of Hartl and Clark^37^, where *F*_ST_-values ranging between 0.05 and 0.15 are considered as moderate genetic differentiation. This appears even smaller, if the 9.17% explained inter-regional variation is compared to the 10-fold higher explained intra-regional variation with 90.73%. Changes in the haplotype composition could not be related to changes in coral performance and tissue composition.

The *Symbiodinium* clade composition of the same coral specimens varied mainly in the most northern and most southern region. ITS2 types of clade C dominating in the Gulf of Aqaba, ITS2 type A1 was present throughout most part of the main Red Sea and the new ITS2 type A21 dominated in the very south (Fig. 5b).

## Discussion

It is undeniable that global change has taken its toll on tropical shallow water coral reefs over the last few decades, already^7^,^24^,^38^. In particular global warming led to losses of coral communities after major bleaching events^39^,^40^,^41^, but also localized eutrophication and sedimentation^7^,^42^. The question whether corals survive these changes, is a questions of whether corals can adjust fast enough and/or whether corals from ‘extreme' conditions (already adjusted) disperse fast enough to regions which become ‘extreme' (e.g. temperatures >31°C over several weeks or even months). In order to approach these questions, we need to know first what the underlying mechanisms of adjustment are by investigating the performance and genetic constitution of corals thriving under naturally differing and extreme thermal (and nutrient) regimes. To the best of our knowledge, this is the first study (i) investigating *in situ* performance and genetic composition of a tropical coral species over such a large latitudinal range (12 latitudes), and (ii) which includes naturally occurring temperatures above 31°C for several weeks or even months per year. We found strong indications for a surprisingly high phenotypic plasticity of the coral *P. verrucosa* despite large distances between reefs and strong environmental gradients in the Red Sea. This was particularly indicated by the strong temperature dependency of calcification independent of temperature history. Acclimatization rather than genetic adaptation is further supported by the low genetic divergence of the coral host from north to south.

Temperature was found to be the best explaining parameter for calcification in *P. verrucosa*, revealing a homogenous response throughout sites. This means that similar calcification rates were found at similar temperatures, independent of prevailing temperature regime at the different sites. If we consider all Red Sea *P. verrucosa* as one population (as genetics indicate, see discussion below) then the calcification performance clearly peaks at 28–29°C. If, in contrast, we consider the local populations as distinct, then the observations that the northern populations show higher calcification rates in summer (maximum temperature), central populations show similarly high rates in summer and winter (minimum and maximum temperatures) and southern populations show higher calcification rates in winter (minimum temperatures) suggests a similar temperature response, i.e. a calcification optimum at an intermediate temperature of 28–29°C. If this would not be the case, we would expect a lower temperature dependency across sites within a given season, meaning similar calcification rates at the different sites, despite different temperatures. In any case, this homogenous response to temperature is surprising considering the low degree of overlap in temperature regimes. In the main Red Sea, there is an overlap of only 2°C between the northern reef 2-WAJ (23–30°C, annual minimum and maximum temperature) and southern reef 5-DOG (28–33°C), and there is even a gap between the temperature regimes of 1-MAQ in the Gulf of Aqaba (21–27.5°C) and 5-DOG^16^. The strong relationship found between calcification and temperature across sites in our study is in contrast to the findings of Carricart-Ganivet^6^. At the same water temperature, Carricart-Ganivet^6^ observed different calcification rates of the coral *Montastrea annularis*, when derived from locations with different temperature regimes (Mexican Gulf: 23.5–29.5°C versus Caribbean: 26.5–29.5°C, max. distance between sites ~1000 km) and concluded that corals are adapted to regional conditions. In contrast to that, a recent study by Rodolfo-Metalpa, et al.^43^ found equal temperature optima for calcification for the temperate coral *Oculina patagonica* from different regions in the Mediterranean and consequently suggested a low regional adaptation of *O. patagonica*. Compared to our study, however, his study included temperature regimes with a much greater overlap between regions, due to high seasonal temperature fluctuations (>10°C), while mean summer temperatures varied up to 3.5°C between sites (max. distance ~3000 km). Considering this, it remains remarkable that calcification of *P. verrucosa* seemingly is not adapted to local temperature conditions in the Red Sea, but rather features a high phenotypic plasticity. (The Gulf of Aqaba with its exceptionally high calcification rates might, however, feature adaptive mechanisms to low temperatures, as discussed further below.) It seems that as long as an optimal temperature window is provided at some point throughout the year no adaptations are required. This, however, would mean that southern corals, which experience optimum conditions only during the coldest season of the year, are particularly vulnerable to global warming.

Besides temperature, light is known to strongly influence calcification rates of zooxanthellate corals, although not directly. This is particularly evident during a circadian cycle, where calcification rates correlate with photosynthetic rates presumably attributable to variable energy supply^17^. This is generally supported by our C-I curves, although with exceptions in summer at 4-JED and 5-DOG, where high summer temperatures (>30°C) and high light intensities (>500 μE m^−2^ s^−1^) co-occurred. These exceptions might be explained by energy allocation towards stress-preventing processes during midday, for example towards the expression and activation of heat-shock proteins^44^. Interestingly, in contrast to 4-JED and 5-DOG, corals from the warmest and most southern reef 6-FAR (~32°C) did not decrease their calcification during midday (Fig. 4), potentially due to the fact that light intensities remained below 500 μE m^−2^ s^−1^ (due to high water turbidity in this nutrient-rich region). In that regard, increased nutrient supply and hence increased turbidity could to some extent mitigate thermal and/or UV stress in shallow reef environments. It is worthwhile mentioning in that context, that coral shading has been proposed as a coral reef management strategy during periods of unusual high water temperatures, previously, in order to reduce additional stress by high UV radiation^45^.

The coral's nutritional condition (biomass, lipid and protein content) and metabolism (photosynthesis, respiration) can significantly influence its calcification and growth^17^,^18^. In return, the nutritional condition is especially dependent on the availability and uptake of inorganic (by zooxanthellae) and organic nutrients (by coral host through heterotrophy) from the water^18^,^26^, and the metabolic activity is dependent inter alia on temperature^46^,^47^. The prevalent variability of nutrient availability and temperature in the Red Sea was therefore assumed to have indirect effects on the coral's calcification rate. Those indirect effects may additionally include processes, which are potentially competing with calcification for energy, such as the highly energy consuming mucus production^48^. Our results revealed an overall rather weak relationship between the environmental conditions and nutritional condition of the corals. The lack of a relationship between biomass and zooxanthellae pigmentation with nutrient availability may be explained by a low capacity for heterotrophy^18^, as suggested for this species before^49^. The positive relationship between tissue protein and lipid content with temperatures but negative relationship with nutrient availability is not trivial. It may, however, be speculated that the increase in temperature, which increased the metabolic activity, resulted in comparatively higher burning rates of carbohydrates than of proteins and lipids and/or in a stronger built up of lipids and proteins compared to carbohydrates.

Although the concentration of photo-collecting pigments could not be related to environmental conditions, it strongly determined the photosynthetic rates, as described in the parallel study on *Symbiodinium* biology of *P. verrucosa*^16^. In that study, a slight temperature dependency of the photosynthetic efficiency (highest between 26 and 28°C) was found, as well^16^. The daily photosynthetic rates, known as the main energy source, in return however, contributed only little to the explained variation in the highly energy consuming calcification rates. And mucus production, an energy consuming process and therefore potentially competing for energy with calcification, could not be related to the calcification rates at all. This is surprising considering that mucus release rates varied substantially, increasing >5-fold from north to south, where it serves as an effective defense barrier against settling dead and living particles and against increased microbial abundance and activity on the coral surface^50^. Also the coral's nutritional conditions contributed only little to the explained variation in calcification rates, leaving temperature as the main driver for calcification. The only additionally explained variation beside temperature was found in summer by the photosynthetic rate and the tissue protein content, which might be due to potentially stressful summer temperatures. Those may lead to increased energy allocation (derived by photosynthesis) into heat stress prevention and cell maintenance (e.g. expression and activity of heat-shock proteins, anti-oxidative proteins, repair proteins)^11^,^44^, consequently leading to lower calcification rates.

Our genetic analyses showed that *P. verrucosa* populations differ only little between the regions along the Red Sea coast, but instead revealed a high intra-regional variation indicating strong panmixia. Although the sample size of our study preclude an exhaustive analyses of inter- and intra-population variation, other studies have also shown small or no regional differentiation for this species along the strong environmental gradients in the Red Sea. Robitzch, et al.^51^ investigated the population structure of *P. verrucosa* based on 360 samples collected between Al-Wajh (2-WAJ) and Doga (5-DOG) with the same mitochondrial marker as used in this study, and with nine additional microsatellite markers. They did not find any differentiation of the coral populations over a distance of 850 km. Preliminary results of another study also did not find a genetic differentiation of *P. verrucosa* throughout the Red Sea, including sites of our study (Banguerra-Hinestroza, unpublished data). Furthermore, a large-scale study on population structures of the genus *Pocillopora* over large geographic regions in the Indo-Pacific also suggest high gene flow and consequently low population subdivisions^52^. These findings together with the absence of a relationship between genetic and physiological constitution may suggest a lack of regional adaptation, which would support a high phenotypic plasticity (e.g. indicated by the strong temperature dependency of calcification across sites). It needs to keep in mind, however, that the lack of genetic divergence at neutral loci does not necessarily mean a lack of genetic divergence elsewhere in the genome, for example at specific, adaption-related loci^53^. Therefore, our results provide indications for, but do not prove yet, low regional adaptation, for which further analyses are necessary.

In contrast to the genetic structure of the coral host, the *Symbiodinium* clade association within the same *P. verrucosa* specimens revealed a slightly different biogeographic pattern^16^. While throughout most parts of the Red Sea clade A1 (*S. microadriaticum*) dominated the symbiosis with *P. verrucosa*, symbiont association differed at the most northern and most southern region (Fig. 5b). In the north (1-MAQ), ITS2 types of clade C prevailed, while in the south (6-FAR), a new ITS2 type, clade A21, dominated^16^. This may indicate acclimatization to prevailing environmental conditions at the extreme ends of the environmental gradient in the Red Sea by promoting association of *P. verrucosa* with different *Symbiodinium* types. The different symbiont types in the north were also found to feature different physiological properties, including a higher photosynthetic efficiency and a lower seasonal regulation of chl *a*/xanthophyll ratios of clade C types compared to clade A1^16^. Given the particularly high calcification rates in the Gulf of Aqaba, we speculate that clade C types may also be able to translocate larger amount of photosynthetically derived energy to the host, thereby boosting calcification rates.

In summary, our study revealed a strong and largely uniform relationship of coral calcification with temperature, despite a strong shift in temperature regime (~6°C) over the 12 degrees in latitude. Independent of latitude and season, highest calcification rates were found between 28°C and 29°C. This suggests a remarkable metabolic plasticity and, at the same time, little regional adaptation, as supported by the low inter-regional genetic divergence. While this may direct to a large capacity to cope with rising water temperatures due to global warming in the northern regions of the Red Sea on one hand, it suggests a limited ability to cope with global warming in the central and particularly in the southern Red Sea. This finding is supported by the high bleaching threshold found for several coral species (not only *Pocillopora*) in the Gulf of Aqaba in relation to prevailing temperatures^54^, and by the observed decelerating coral growth rates in the central Red Sea as a consequence of increasing water temperatures over the last two decades^3^. Red Sea coral genotypes featuring a high phenotypic plasticity and thermo-tolerance might therefore be able to sustain diverse and complex coral communities in the northern Red Sea during ocean warming and may even be considered as a ‘genetic reservoir' to restock other biogeographic regions, where ocean warming decimate local coral communities. However, there seem to be upper thermal limits for physiological performance as well as adaption, considering that corals originating from the central and southern Red Sea perform better at ‘cold' winter temperatures than at ‘hot' summer temperatures.

## Supplementary Material

Supplementary Information Supplementary Information

## Figures and Tables

**Table 1:** Result of multiple regression analyses. Response variables are presented underlined, predictor variables are temperature and ‘nutrients'. Nutrients are inferred from the concentration of water chlorophyll *a* multiplied by the relative water flow and values are derived from Sawall, et al.^16^. Temperature and ‘nutrients' are independent of each other (r^2^ = 0.16, p > 0.05)

Response variable:	β	SE of β	B	SE of B	t	p
**Biomass cm^−2^**(Adjusted R^2^ = ---; F(2.61) = 0.479; p = 0.621)						
Temperature	−0.118	0.129	−0.048	0.052	−0.921	0.360
‘Nutrients'	0.062	0.129	0.065	0.135	0.480	0.633
**Protein, % of biomass** (Adjusted R^2^ = 0.207; F(2.61) = 9.021; p = 0.000)						
Temperature	0.401	0.114	1.093	0.310	3.521	**0.001**
‘Nutrients'	−0.342	0.114	−2.426	0.808	−3.003	**0.004**
**Lipid, % of biomass** (Adjusted R^2^ = 0.566; F(2.61) = 42.152; p = 0.000)						
Temperature	0.647	0.084	0.320	0.042	7.670	**0.000**
‘Nutrients'	−0.522	0.084	−0.671	0.108	−6.207	**0.000**
**Photo-collecting pigments cm^−2^** (Adjusted R^2^ = ---; F(2.61) = 0.076; p = 0.927)						
Temperature	−0.009	0.130	−0.011	0.151	−0.072	0.943
‘Nutrients'	0.051	0.130	0.153	0.392	0.390	0.700
**Mucus release cm^−2^ d^−1^** (Adjusted R^2^ = 0.707; F(2.61) = 77.061; p = 0.000)						
Temperature	0.554	0.069	0.162	0.020	8,011	**0.000**
‘Nutrients'	0.555	0.069	0.423	0.053	8,032	**0.000**

**Table 2:** Results of distance based linear models (DistLM). Response variable is calcification and predictor variables are temperature, mucus release, photosynthesis, biomass and % protein and % lipids of biomass. Only the best fitting models are presented

Response variable: Calcification cm^−2^ d^−1^						
Season:	Adj. R^2^	SS	Pseudo-F	p	Probability	res. df
**September & March**						
Temperature	0.447	4725	9.880	**0.003**	0.497	10
Mucus release cm^−2^ d^−1^	0.544	1236	3.135	0.098	0.130	9
Lipids, % of biomass	0.610	851	2.524	0.137	0.089	8
**September**						
Temperature	0.656	308	10.538	**0.012**	0.725	4
Protein, % of biomass	0.803	67	3.994	**0.023**	0.157	3
Photosynthesis cm^−2^ d^−1^	0.948	41	9.369	**0.041**	0.097	2
Lipids, % of biomass	0.962	6	1.802	0.320	0.013	1
**March**						
Temperature	0.781	4091	18.879	**0.015**	0.825	4
Biomass cm^−2^	0.831	364	2.169	0.166	0.073	3
Protein, % of biomass	0.919	342	4.261	0.145	0.069	2
Mucus release cm^−2^ d^−1^	0.984	145	8.973	0.169	0.029	1
