# Supplementary material for: Extensive phenotypic plasticity of a Red Sea coral over a strong latitudinal temperature gradient suggests limited acclimatization potential to warming
Source: Sci Rep. 2015 Mar 10;5:8940. doi: 10.1038/srep08940 (PMC5155415; doi:10.1038/srep08940)
Supplement: Supplementary Information [file srep08940-s1.pdf]

Extensive phenotypic plasticity of a Red Sea coral over a strong latitudinal temperature gradient suggests limited acclimatization potential to warming

Yvonne Sawall, Abdulmoshin Al-Sofyani, Sönke Hohn, Eulalia Banguera-Hinestroza, Christian R. Voolstra, Martin Wahl

Supplementary methods

Calculation of daily metabolic rates (photosynthesis, respiration, calcification, mucus release)

Gross oxygen production (*P*) and oxygen consumption rates (*R*) were converted to carbon gain and loss (CO_2) rates assuming the metabolic quotients PQ (gross photosynthetic quotient) with 1.1 mol O2 : 1 mol CO_2 and RQ (respiratory quotient) with 0.8 mol CO_2 : 1 mol O_2 (Muscatine *et al.*, 1981, Tremblay *et al.*, 2012). There are only very few studies presenting metabolic quotients for corals (Gattuso & Jaubert, 1990, Muscatine *et al.*, 1981), which is particularly difficult to determine in animal-plant symbiotic relationships. Those values vary dependent on environmental conditions (e.g. light availability (Gattuso & Jaubert, 1990)) and substrate consumed or produced (lipids, glucose or proteins) by the zooxanthellae and coral host (Gattuso & Jaubert, 1988, Gnaiger, 1983). We chose the values 1.1 for PQ and 0.8 for RQ, as they were previously calculated and/or found suitable for corals living in 1-5 m depth of two coral species belonging to the same coral family (*Stylophora pistillata* (Gattuso & Jaubert, 1990, Tremblay *et al.*, 2012) and *Pocillopora damicornis* (Muscatine et al., 1981)) as our study species *P. verrucosa*.

Calcification (*C*) and mucus release (*M*) rates were already measured as carbon precipitation ($CaCO_3$) and carbon release (particulate carbon) rates, respectively, therefore no conversion was necessary.

Prior the calculation of daily rates of *P* and *C*, idealistic light intensity curves over 24h were constructed for each site and season (*i*), using the corresponding maximum measured PAR at noon (PAR_{noon}) during experimentation and assuming a 12:12h day:night cycle (day length, *dl* =12). The apparent photosynthetic available radiation (PAR) over a time period of 24 hours using a one second interval (*t*) was calculated for all six sites and both seasons (*i*=1 to 12). The parameter '*width*' in the equation determines the irradiance at dusk and dawn and is chosen (width=0.3) to result approximately 6% of maximum irradiance at sunrise or sundown.

$$PAR(t,i) = 1 + PAR_{noon}(i) \cdot e^{\left(\frac{(-hour(t)-12)^2}{(dl \cdot width)^2}\right)}$$

The simulated diurnal cycles of PAR at all sampling sites and during both sampling periods are presented in Supplementary Fig. S3.

The measured data points of the P-I and C-I curves (Supplementary Fig. S1) were fitted to the idealistic light intensity curves and daily rates were calculated via integration. The calculated rates are then integrated over the simulation period to obtain total daily *P* and *C* rates, respectively.

$$P(t,i) = P_{\max}(i) \cdot \left(1 - e^{-\alpha(i) \cdot PAR(t,i)}\right)$$

$$C(t,i) = C_{\max}(i) \cdot \left(1 - e^{-\beta(i) \cdot PAR(t,i)}\right) \cdot e^{-\gamma(i) \cdot PAR(t,i)}$$

The simulated P-I and C-I curves are presented in Supplementary Fig. S2.

Daily rates of *M* were calculated assuming a 75% reduction of *M* during the night (Naumann *et al.*, 2010) (= 25% of day time *M*). Daytime mucus release then increases as a function of the photosynthetic carbon fixation rate (*P*) to 100% of the measured rates.

$$M(t,i) = M_{\max}(i) \cdot 0.25 + \frac{P(t,i)}{P_{\max}(i)} \cdot M_{\max}(i) \cdot 0.75$$

Daily rates of *R* were calculated similarly to *M*, assuming enhanced *R* of 58% during the day (Edmunds & Davies, 1988), due to the fact that increased substrate and O_2 is provided for *R* through photosynthetic carbon fixation (McCloskey & Muscatine, 1984).

$$R(t,i) = \frac{R_{\max}(i)}{1.58} + \frac{P(t,i)}{P_{\max}(i)} \cdot \frac{R_{\max}(i)}{1.58} \cdot 0.58$$

References

Edmunds PJ, Davies PS (1988) Post-illumination stimulation of respiration rate in the coral *Porites porites*. *Coral Reefs,* **7**, 7-9.

Gattuso J-P, Jaubert J (1988) Computation of metabolic quotients in plant-animal symbiotic units. *Journal of Theoretical Biology,* **130**, 205-212.

Gattuso J-P, Jaubert J (1990) Effect of light on oxygen and carbon dioxide fluxes and on metabolic quotients measured in situ in a zooxanthellate coral. *Limnology and Oceanography,* **35**, 1796-1804.

Gnaiger E (1983) Calculation of Energetic and Biochemical Equivalents of Respiratory Oxygen Consumption. In: *Polarographic Oxygen Sensors.* (eds Gnaiger E, Forstner H) pp Page., Springer Berlin Heidelberg.

Mccloskey LR, Muscatine L (1984) Production and respiration in the Red Sea coral *Stylophora pistillata* as a function of depth. *Proceedings of the Royal Society of London Series B-Biological Sciences,* **222**, 215-230.

Muscatine L, Mccloskey LR, Marian RE (1981) Estimating the daily contribution of carbon from zooxanthellae to coral animal respiration. *Limnology and Oceanography,* **26**, 601-611.

Naumann M, Haas A, Struck U, Mayr C, El-Zibdah M, Wild C (2010) Organic matter release by dominant hermatypic corals of the Northern Red Sea. *Coral Reefs,* **29**, 649-659.

Tremblay P, Grover R, Maguer JF, Legendre L, Ferrier-Pagès C (2012) Autotrophic carbon budget in coral tissue: a new 13C-based model of photosynthate translocation. *J Exp Biol,* **215**, 1384-1393.

Extensive phenotypic plasticity of a Red Sea coral over a strong latitudinal temperature gradient suggests limited acclimatization potential to warming

Yvonne Sawall, Abdulmoshin Al-Sofyani, Sönke Hohn, Eulalia Banguera-Hinestroza, Christian R. Voolstra, Martin Wahl

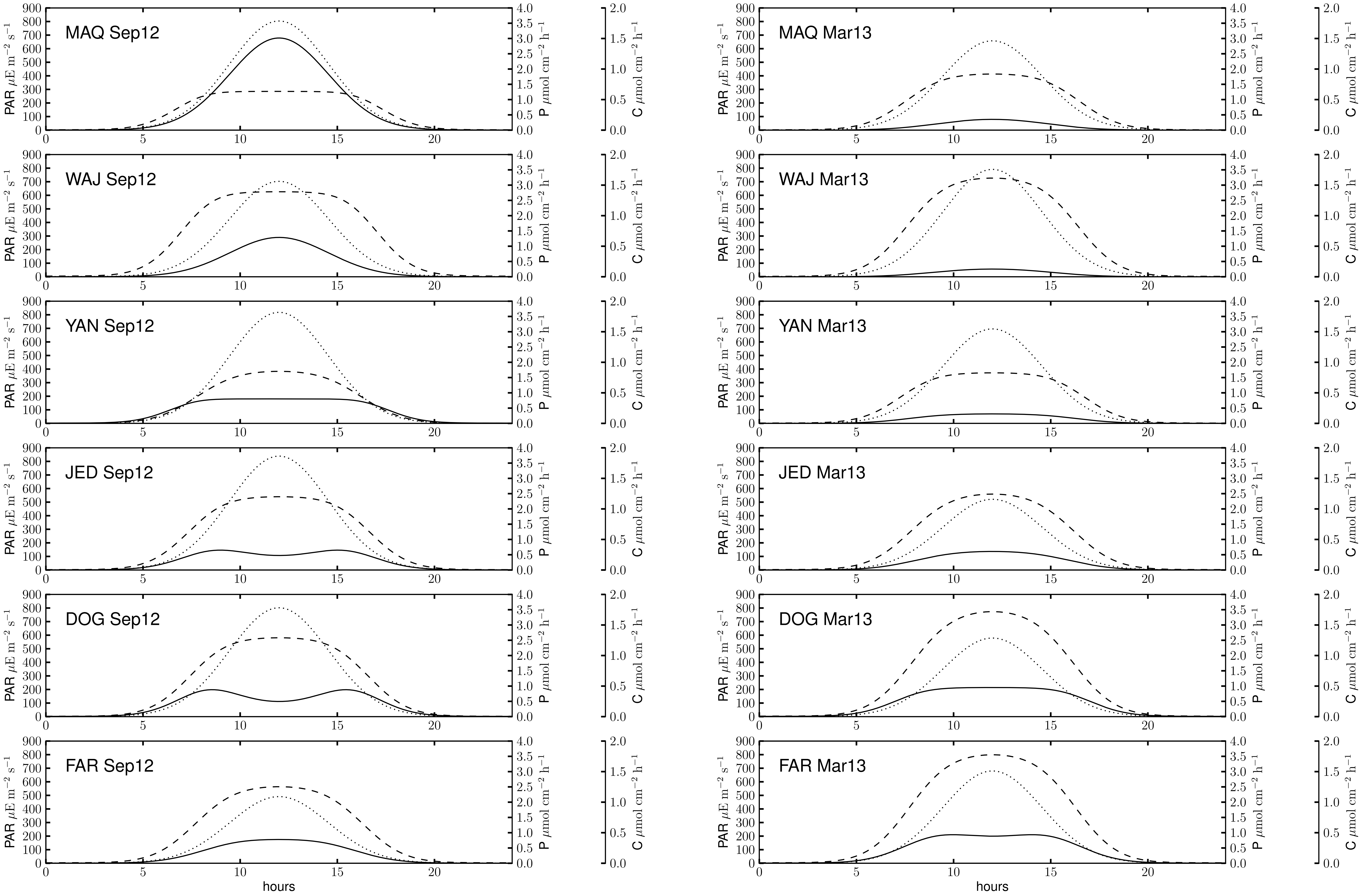

Fig. S1: Reconstructed daily curves (24h) of photosynthetic active radiation (PAR, dotted line), photosynthesis (dashed line), and calcification (straight line).

Extensive phenotypic plasticity of a Red Sea coral over a strong latitudinal temperature gradient suggests limited acclimatization potential to warming

Yvonne Sawall, Abdulmoshin Al-Sofyani, Sönke Hohn, Eulalia Banguera-Hinestroza, Christian R. Voolstra, Martin Wahl

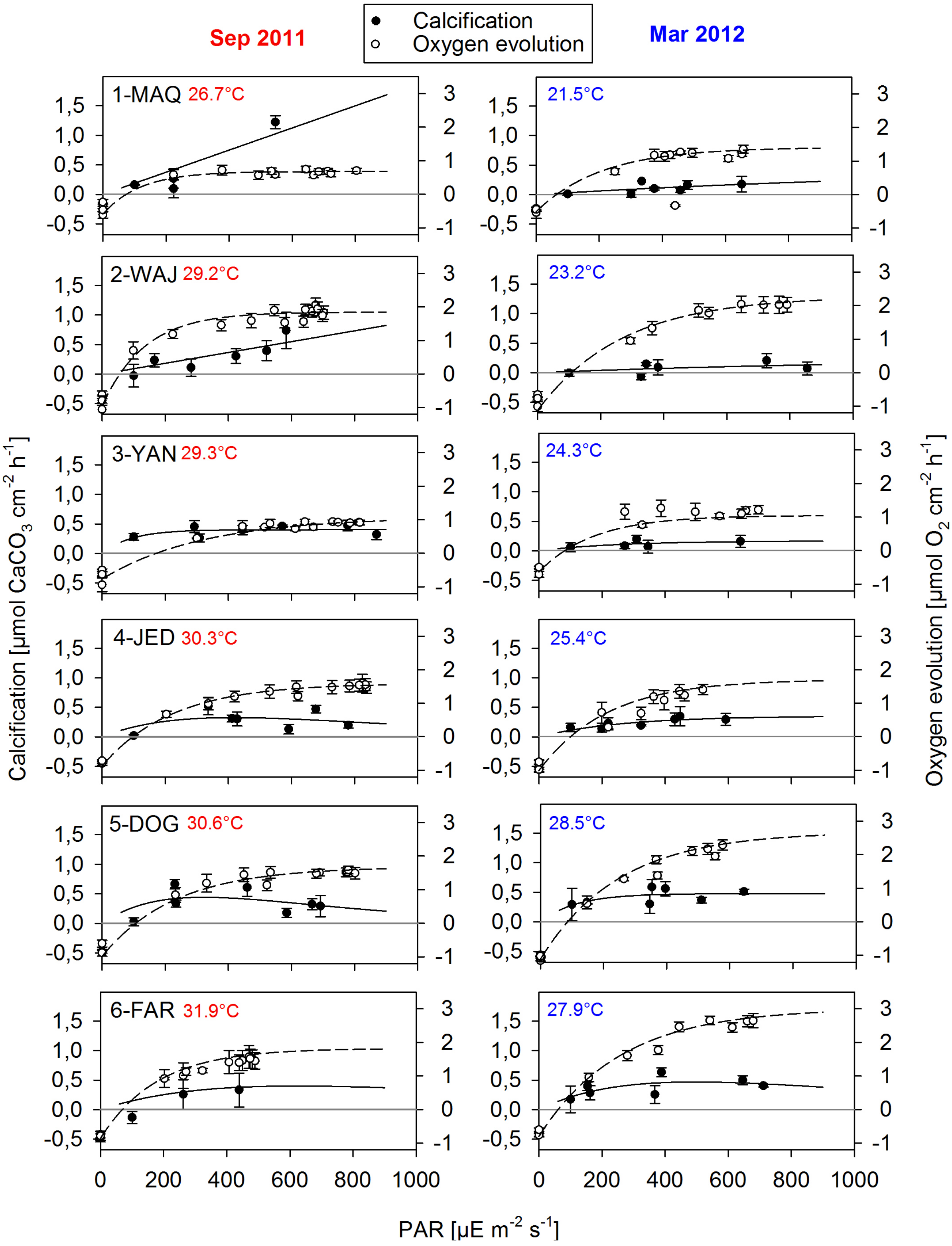

Fig. S2: Comparison of calcification (black) and oxygen evolution (white) versus light curves at all sites from N (top) to S (bottom) in September 2011 (left) and March 2012 (right). Mean ± SE (n=3-6). Mean monthly temperatures at the experimental depth of 5 m are indicated in red (September 2011) and blue (March 2012). P-I curves were previously published in Sawall, et al. (16).

Extensive phenotypic plasticity of a Red Sea coral over a strong latitudinal temperature gradient suggests limited acclimatization potential to warming

Yvonne Sawall, Abdulmoshin Al-Sofyani, Sönke Hohn, Eulalia Banguera-Hinestroza, Christian R. Voolstra, Martin Wahl

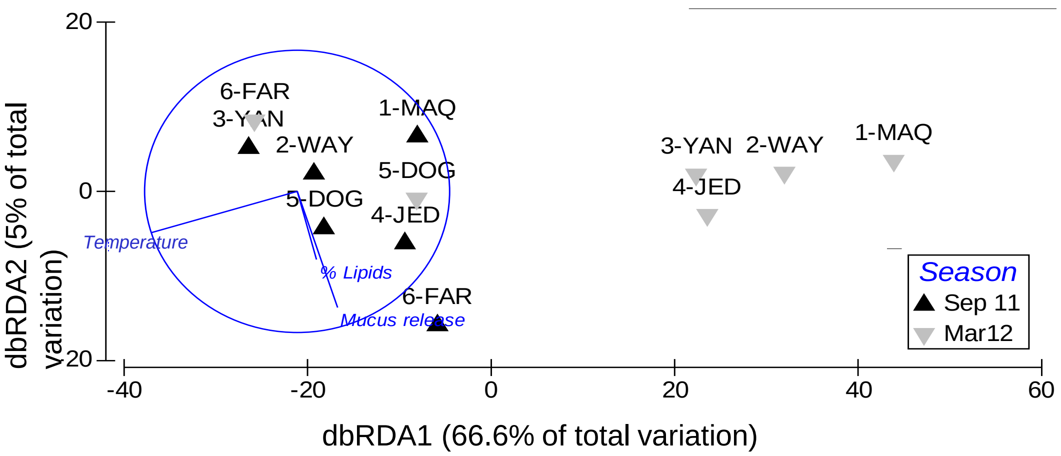

Fig. S3: Results of distance based redundancy analysis (dbRDA). Triangles represent the calcification (response variable) at a given location and season, distance between triangles is based on a Bray-Curtis similarity matrix, vectors represent the direction and strength (length of vector) of the predictor variables. Corresponding distance-based linear model (DistLM) results are provided in Tab. 2 - September & March.
